# Development of a Sensitive ELISA for Gastric Intrinsic Factor and Detection of Intrinsic Factor Immunoreactivity in Human Serum

**DOI:** 10.3390/nu14194043

**Published:** 2022-09-28

**Authors:** Eva Greibe, Ebba Nexo

**Affiliations:** 1 Department of Clinical Biochemistry, Aarhus University Hospital, 8200 Aarhus, Denmark; 2 Department of Clinical Medicine, Aarhus University, 8000 Aarhus, Denmark

**Keywords:** intrinsic factor, ELISA, vitamin B12, cobalamin, pernicious anemia, autoimmune disease

## Abstract

Gastric Intrinsic Factor (IF) is produced by the parietal cells of the stomach and secreted into the gastrointestinal tract where it ensures the active absorption of vitamin B12. We hypothesized that a small amount of IF ends up in the circulation and can be measured in serum. The aim of this study was to develop an assay for measuring human IF and to demonstrate its presence in serum. We designed a sensitive ELISA for measurement of human IF using a commercial monoclonal antibody and an in-house polyclonal antibody as capture and detecting antibody, respectively. Imprecision, accuracy, and linearity of the assay were examined. We established a reference interval based on serum samples from 240 healthy donors, and explored the daily IF fluctuations in 20 healthy subjects. Employing a prototype IF ELISA and size exclusion chromatography experiments, we demonstrated the presence of IF in human serum. In its final design, the IF ELISA has a measurement range of 0.2 to 50 pmol/L. The intra-assay and total imprecision were 7.9% and 15%, respectively. The 95% reference interval (18–65 years) was 1.7–11.6 pmol/L. No diurnal fluctuation or notable sex differences were observed. Our results suggest that the assay is capable of detecting and quantifying human IF in the circulation and may prove useful in the characterization of patients with impaired IF production.

## 1. Introduction

The protein Gastric Intrinsic Factor (IF) is produced by parietal cells in the gastric mucosa and secreted into the gastrointestinal tract where it facilitates the active intestinal absorption of vitamin B12 (B12) [1]. Patients with the autoimmune disease, pernicious anemia, have lost the ability to produce IF and thereby the ability to absorb B12 via active IF-mediated transportation [1,2]. 

IF has been identified in gastric juice in concentrations of nmol/L [3,4], but measures of the protein in gastric juice is of little clinical use due to difficulties in persuading patients to donate sample material and due to difficulties with standardization of samples collection. 

Other exocrine secreted proteins, such as salivary amylase, have also been shown to occur in minute amounts in the circulation where measures of the protein have proven to be clinically useful [5]. 

So far, no one has identified IF in the circulation, possibly due to the lack of an assay sufficiently sensitive to detect minute amounts of the protein. Gräsbeck et al. (1982) developed a radioimmunoassay for IF detection and applied it on urine and serum, but was unable to detect IF in serum [4]. 

In the present paper, we describe the development of a sensitive method for measurement of IF and show that the protein is present in human serum in healthy adults.

## 2. Materials and Methods

For our IF assay, we employed a standard sandwich ELISA design using an immobilized antibody to capture IF and a second biotinylated detection antibody that reacts with horseradish peroxidase-avidin, producing a color reaction proportional to the IF concentration. 

We used a prototype ELISA to verify the presence of IF in human serum (proof of concept). The reagents and detailed assay procedure for both prototype ELISA and final ELISA are outlined below together with the methods for assay validation and measurement on serum from healthy controls.

### 2.1. Antibodies, Calibrators, and Controls

Two polyclonal rabbit anti human IF antibodies were custom-made towards native pure human IF [6] by immunization of two rabbits (F2831 and F2832) (DAKO A/S) followed by purification of the γ-globulin fraction of the rabbit serum, as previously described [7]. The freeze-dried rabbit F2831 γ-globulin 0994 was biotinylated by coupling active biotin to the antibody and inactivating excess biotin with lysine, as described in [7], and used for detecting antibody. For capturing antibody, we used freeze-dried rabbit F2832 γ-globulin 0994 for the prototype ELISA, and a monoclonal mouse anti human IF antibody (Bio-rad Laboratories, Copenhagen, Denmark, cat. no. MCA5886G) for the final ELISA assay. 

Recombinant human IF was used as calibrator by dissolving 20 nmol/mL apo-IF (Cobento Biotech A/S, Aarhus, Denmark) in 0.1% phosphate buffered albumin (PBA) made by dissolving bovine serum albumin (Sigma-Aldrich, Soeborg, Denmark, cat. no. A7030) in a 0.1 M phosphate buffer (VWR International, Soeborg, Denmark, cat. no. AMPQ40654), pH 8. The calibrators were prepared to cover a concentration range of 0.8 to 50 pmol/L by dilution in assay buffer (assay buffer details are given below), which was also used as a zero calibrator. All calibrators were stored at −20 °C until used.

Two levels of control sera were prepared. A low control (IF concentration of 4 pmol/L) was made from a pool of anonymous donor serum that was centrifuged for 9 min at 1850× *g* at room temperature. A high control (IF concentration of 30 pmol/L) was made by spiking 39.8 mL of the same serum pool with 122 µL depepsinated gastric juice. The gastric juice was collected by medical staff as part of a diagnostic test for acidity (Bispebjerg Hospital, Copenhagen, Denmark). Excess samples of gastric juice—used in this study—were collected with no information allowing the samples to be traced back to the donor. After preparation, the two controls were aliquoted and stored at −20 °C until usage. When running the ELISA, the high and low controls were placed both before and after the samples on the microtiter plate.

### 2.2. The ELISA Procedure

ELISA microtiter plates (Nunc F96 Maxisorp 442404, Thermo Scientific, Odense, Denmark) were coated with 1-µg anti-IF catching antibody as described in [7]. The coated plates were stored at −20 °C and removed to room temperature one hour before usage.

The prototype ELISA was conducted as outlined below for the final ELISA assay, except for (i) using a polyclonal rabbit antibody for capturing reagent; (ii) incubating the samples overnight at 4 °C; and (iii) incubating with detection antibody for 3 h at room temperature.

The final ELISA assay was conducted as follows: The plates were washed three times in 350 µL washing buffer (VWR International, Soeborg, Denmark, cat. no. AMPQ15265) containing 1.5 mM NaH_2_PO_4_, 8.5 mM Na_2_HPO_4_, 145 mM NaCl, 1 g/L Tween 20, and demineralized H2O, pH 7.4, by the Biotec ELx50 microtiter plate washer (Holm & Halby A/S, Broendby, Denmark). Then, 50-µL assay buffer was added to each well. The assay buffer was prepared by adding B12 (cyanocobalamin, Sigma-Aldrich, cat. no. V2876) to 0.1% PBA to a final concentration of 1 nM B12. The purpose of adding B12 to the buffer was to ensure that all IF was saturated with B12 before analysis to ensure that all IF molecules were present in the same molecular form, IF saturated with B12. Next, 50-µL sample or 50-µL control was added to the wells. As the calibrators are prepared by dilution in the assay buffer (see details above), 100 µL calibrators are added directly to the wells (without pre-addition of 50 µL assay buffer). After incubation for 2.5 h at room temperature with gentle shaking, the plates were washed three times in washing buffer. The detection antibody was diluted 1:500 to a concentration of 0.868 mg/mL in 0.1% PBA (without B12) and 100 µL of this solution was added to each well, 0.174 µg/well, and incubated for 2 h at room temperature with gentle shaking. The plates were then washed three times again in washing buffer before addition of 100 µL of horseradish peroxidase-avidin to each well. This reagent contained 6 µL of avidin-peroxidase-conjugate (Sigma-Aldrich, cat. no. A7419) diluted 1:30, 120 µL lysozymes, and 12 mL POD buffer (VWR International, cat. no. AMPQ42067) pH 7.4 made from 1.5 mM NaH_2_PO_4_, 8.5 mM Na_2_HPO_4_, and 400 mM NaCl in demineralized water. The plates incubated 30 min with the POD reagent at room temperature under gentle shaking. The plates were then washed three times in washing buffer before addition of color reagent; 100 µL TMB ONE Ready-to-use Substrate (KEM EN TEC, Taastrup, Denmark, cat. no. 4380A) to each well. The plates were incubated for 13–14 min at room temperature during gentle shaking before the color reaction was stopped by adding of 100-µL of 0.1 M phosphoric acid made by diluting 136.3 mL of 85% orthophosphoric acid (VWR International, cat. no. 20621) in 2 L ELGA water. By photometry, the color development was measured at 450 nm and corrected for absorbance at 620 nm on the MultiSkan Ascent (Thermo Scientific, Odense, Denmark). The calibration curve was computed by plotting the absorbance of the calibrators and constructing a cubic spline curve with linear scale on both axes. The results for samples and controls were read from this curve. 

### 2.3. Accuracy and Correctness 

To test analytical interference from human transcobalamin (TC) and haptocorrin (HC), the two known B12-binding proteins present in the serum [1], solutions with high concentrations of human TC (50 nM ApoTC, a kind gift from Sergey Fedosov) and human HC (60 nM derived from a serum samples with exceptional high concentration of ApoHC [8]) in 0.1% PBA was prepared and analyzed with the IF ELISA assay, together with the calibrators and controls. 

To confirm that the ELISA signal observed in serum was human IF, size exclusion chromatography studies were performed. Human serum (500 µL) was applied to a Superdex^®^ 200 HR 10/30 column (GE Healthcare, Broendby, Denmark) on a Dionex^®^ ICS-3000 HPLC system (Dionex Corporation, Hvidovre, Denmark). Blue Dextran (Sigma-Aldrich) and Na22 (GE Healthcare) was used for determination of void volume (V0) and total volume (Vt), respectively. Collected fractions (400 µL/min) were analyzed for concentrations of IF by the prototype ELISA. For comparison, size exclusion chromatography of the human B12-binding proteins, TC and HC, was performed. To further test the accuracy of the IF ELISA assay, we dissolved and analyzed a commercially available stock of human IF (10 µg) (Prospec, East Brunswick, USA) in four different concentrations (10, 25, 40, and 50 pmol/L).

### 2.4. Linearity, Imprecision, Recovery, and Stability

The final IF ELISA assay was validated by the following methods: 

To study linearity, two serum stocks with high (26 pmol/L) and low (3.3 pmol/L) concentration of IF were pooled to give the expected IF concentrations of 26, 21, 17, 12, 7.8, and 3.3 pmol/L. This was done by mixing: 100% high control + 0% low control (26 pmol/L), 80% high control + 20% low control (21 pmol/L), 60% high control + 40% low control (17 pmol/L), 40% high control + 60% low control (12 pmol/L), 20% high control + 80% low control (7.8 pmol/L), and 0% high control + 100% low control (3.3 pmol/L). These pools (serial dilutions) were run in quadruples on each of six days over a period of two months (giving a total of 24 runs per pool). The measured values were plotted against the expected values to assess linearity. The intra-assay imprecision (CV%intra) (variation within the same microtiter plate) was calculated for each pool in each of the six runs by finding the standard deviation of the quadruple results, dividing that by the quadruple mean and multiplying by 100. The mean intra-assay imprecision for each pool was determined by taking the average of the six individual CVs (one determined for each run).

For determination of total imprecision (CV%total), controls in two IF concentrations (4 pmol/L and 30 pmol/L) were analyzed over approx. three year (January 2019–February 2022), altogether 65 times. The two controls were analyzed in duplicate in 31 runs and the double determinations were used to calculate the overall intra-assay imprecision (CV%intra) (variation within the same microtiter plate) of the analysis. Two control batches were employed with no variation in mean value for the low control but with a minor difference for the high control (28.2 versus 31.7 pmol/L), which was not taken into consideration for the further analyses.

Limit of Blank (LoB) was defined as the highest apparent analyte concentration expected to be found when replicates of a blank samples containing no analyte are tested, as described by [9]. The LoB was determined from analysis of 32 blank samples and calculated as follows: LoB = mean_blank_ + 1.645 × SD_blank_. Limit of Detection (LoD) was defined as the lowest analyte concentration likely to be reliable distinguished from the LoB and at which detection is feasible, as described by [9]. The LoD was determined from the LoB and analysis of 23 low concentration samples (1 pmol/L) and calculated as follows: LoD = LoB + 1.645 × SD_low concentration sample_.

Recovery was determined on each of the 20 serum pools with mean IF concentration of 14 pmol/L made from pooling of 40% of the low control and 60% of the high control. Recovery was defined as: (final concentration—initial concentration (low control))/added concentration (high control). The overall recovery was calculated by taking the average of the recovery of the 20 serum pools.

To test the stability of IF in serum samples, we performed eight freeze-thaw cycles with four pools of fresh (not previously frozen) human donor serum. For each freeze-thaw cycle, we analyzed the four different pools with the IF ELISA together with a newly thawed aliquot of the same four pools. The frozen samples were stored at −20 °C and analyzed over a period of 1 week to 15 weeks. After 33 months, the samples were reanalyzed for evaluation of long-term storage.

### 2.5. Measurement on Blood from Healthy Subjects

For establishment of an IF reference interval, blood samples collected in serum tubes (BD Vacutainer^®^, Becton Dickinson, Herlev, Denmark) were obtained from 240 healthy volunteer blood donors at Aarhus University Hospital, Denmark, in January 2022 (*n* = 60, men aged 18–40 years; *n* = 60, men aged 41–65 years; *n* = 60, women aged 18–40 years; and *n* = 60, women aged 41–65 years). The samples were immediately anonymized upon collection, and centrifuged (10 min at 2300× *g*) within four hours. Serum was stored at −20 °C for later analysis of IF as described above for the final ELISA assay. No ethical approval was necessary according to national law.

For investigation of daily fluctuations in serum IF, healthy individuals were recruited by advertisement at Aarhus University Hospital in Denmark in summer 2018. In total, 21 healthy Danish individuals aged ≥18 years were included in the study. Most of them were staff at the hospital. Exclusion criteria was any known chronic systemic disease. The study was performed within the confines of the Helsinki Declaration II, and the study was approved by the Central Denmark Region Ethics Committee (project no. 1-10-72-452-17). All individuals gave their informed consent before inclusion in the study. Blood was drawn on Day 1 (9 o’clock), Day 2 (9, 12, 15, 18, and 21 o’clock), and on Day 3 (9 o’clock) (24-h clock format), and centrifuged (10 min at 2300× *g*) within two hours. Serum was stored at −20 °C for later analysis of IF as described above for the prototype ELISA. One participant showed spurious high serum IF levels (1755 pmol/L) and was removed from the dataset as an outlier.

### 2.6. Statistical Analyses

Data were tested for normality using Shapiro-Wilk’s test as well as assessed using histograms and quantile-quantile plots. Reference intervals were established according to the Clinical and Laboratory Standards Institute (CLSI) approved Guideline “C18-A3; Defining, Establishing, and Verifying Reference Intervals in the Clinical Laboratory” [10]. Reference Intervals (2.5th and 97.5th percentile) were calculated based on the non-parametric method. The Mann-Whitney test (not normally distributed data) was used to compare study groups. To compare daily fluctuations in serum, the repeated measurements analysis of variance (RM-ANOVA) was used. As the overall ANOVA analysis was negative, no post hoc testing between time points was performed. Values of *p* ≤ 0.05 were accepted as statistically significant unless otherwise stated. The data analysis was performed while using the statistical software (San Diego, CA, USA) available in GraphPad Prism version 7.03 and Analyze-It 4.65.3 software for Microsoft Excel (Redmond, WA, USA).

## 3. Results

### 3.1. Proof of Concept

**Figure 1:**
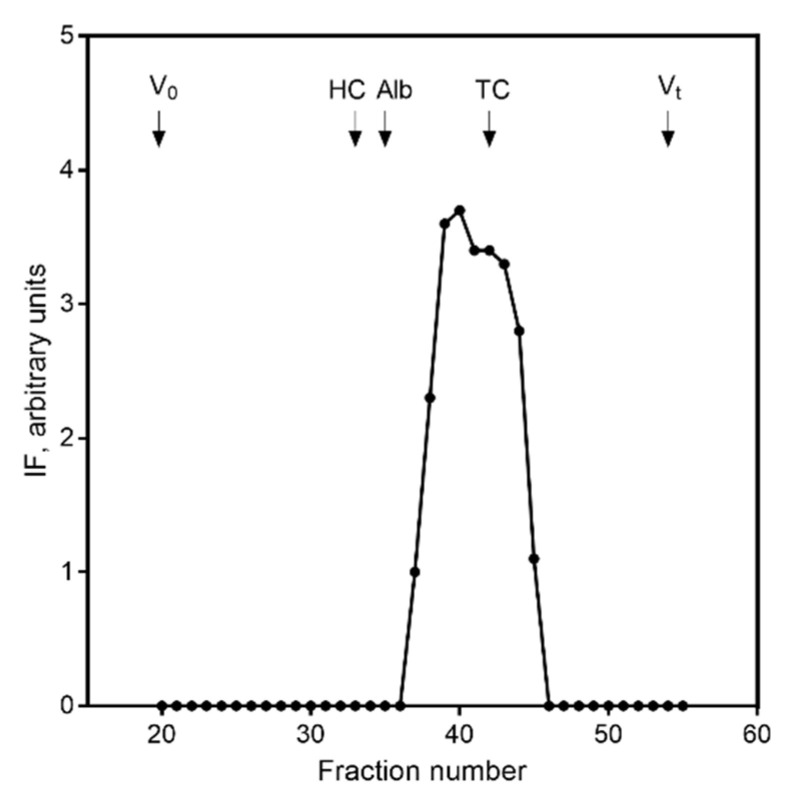
Presence of human IF in serum. Size exclusion chromatography of endogenous human IF in serum measured by prototype IF ELISA. X-axis indicates the fraction number. Human IF eluted in fraction 40. Elution volume for void volume (V0) (fraction 20), human HC (fraction 33), albumin (fraction 35), human TC (fraction 42), and total volume (Vt) (fraction 54) are shown by arrows for comparison. Results are given in arbitrary units.

Initially, we developed a prototype ELISA for human IF based on our previous experience from comparable ELISAs for other B12 binding proteins [11,12]. We used two polyclonal antibodies against human IF as the catching and detection reagent for measurement of IF in human serum. We used this assay to prove the presence of ELISA reactivity in human serum and to verify the IF reactivity eluted as does human IF upon size exclusion chromatography (Figure 1). We interpret this result as indicating the presence of IF in serum. The results from the size exclusion chromatography did not suggest any cross reactivity with the two well characterized B12-binding protein, TC and HC, both eluting differently from IF and present in serum in concentrations of around 0.5–1.5 nmol/L [1]. The results were confirmed by testing high concentrations of TC (50 nM) and HC (60 nM). Neither resulted in any signal in the ELISA (data not shown). This supported that the assay was not influenced by the presence of TC and HC in the serum.

### 3.2. Final Assay Design and Assay Validation

Based on our proof of concept indicating the presence of IF in serum, we decided to optimize the assay in order to perform rigid validation and establish an interval of reference.

**Figure 2:**
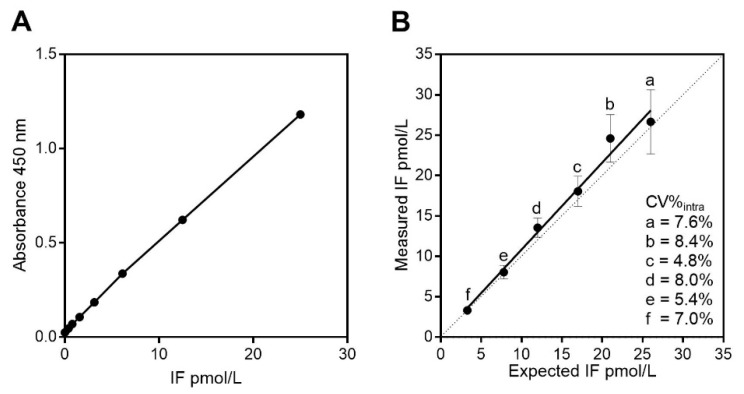
Evaluation of the ELISA for human IF on serum. (**A**) A typical calibration curve for IF in the concentration range of 0.2–50 pmol/L. (**B**) Linearity for the IF ELISA. Serum pools with high (a = 26 pmol/L) and with low (f = 3.3 pmol/L) levels of IF as determined by the IF ELISA were mixed together to get the expected IF concentrations of 21 (b), 17 (c), 12 (d), 7.8 (e) pmol/L. Levels of IF were measured 24 times over six days by the IF ELISA and results measured were plotted as mean with SD against the expected values. The mean intra-assay (within plate) imprecision (CV%intra) calculated from each serum pool is indicated. Linearity for measurement of serum IF showed only minor deviations from linearity (r^2^ = 0.99) and the slope and intercept of the linear regression line (y=1.08x + 0.062) did not deviate significantly from 1 and 0, respectively.

Recombinant IF was employed as calibrator, and a standard calibration curve is depicted in Figure 2A. The optimized assay has a low non-specific binding with a mean absorbance at OD 450 nm of 0.019 (*n* = 32 blank samples). The LoB was calculated to be 0.029 pmol/L. Based on the LoB and SD for samples (*n* = 23) containing 1 pmol/L IF (SD = 0.098 pmol/L), the LoD was found to be 0.189 pmol/L (see method section for calculations of LoB and LoD). The IF ELISA showed a good linearity (r^2^ = 0.99) (Figure 2B). The total imprecision (CV%total) was 16.8% at 4 pmol/L and 13.3% at 30 pmol/L based on 65 measures performed in 34 analytical runs over three years with a mean total imprecision of 15%. We observed no drift in the values obtained underscoring the stability of IF upon storage. The intra-assay imprecision (CV%intra) (within plate) was calculated based on duplicate measures in 31 runs and was 8.0% at 4 pmol/L and 7.8% at 30 pmol/L with a mean overall intra-assay imprecision of 7.9%.

The recovery was judged from analyzing 20 spiked samples and ranged from 72% to 93% (mean 85%) for a mean IF concentration of 14 pmol/L. Accuracy was also tested by analyzing a commercially available stock of human IF (10 µg) in four different concentrations (10, 25, 40, 50 pmol/L). Despite high dilutions factors, the mean accuracy was 20% of the nominal concentrations, which in general is considered acceptable for ligand binding assay [13].

When testing sample stability, the serum IF concentration varied between 1.6% and 8.5% for the four serum samples (serum IF from 5–10 pmol/L) through eight freeze-thaw cycles over a period of 15 weeks. No systematic decline or increase of the concentration was seen (data not shown). After 33 months, the four serum samples were reanalyzed for evaluation of stability after long-term storage. We found the IF concentration to be between 12% and 16% higher than baseline, which is within common acceptable criteria (±20%) for stability data on ligand binding assays [13].

### 3.3. Measurement of Serum IF from Healthy Individuals

To establish an interval of reference, we analyzed serum from 240 healthy volunteer blood donors (*n* = 60, men aged 18–40 years; *n* = 60, men aged 41–65 years; *n* = 60, women aged 18–40 years; and *n* = 60, women aged 41–65 years). We found a reference interval of (2.5th–97.5th percentile with (90%CI)) with a lower limit of 1.7 (0.9; 1.9) pmol and an upper limit of 11.6 (10.0; 16.5) pmol/L (*n* = 240). The 2.5th and 97.5th percentiles when divided according to age and sex are shown in Table 1. We found slightly higher median IF concentrations in middle-aged subjects (41–65 years) (median (range)) (5.1 (1.7–13.8) pmol/L) (*n* = 120) compared with younger (18–40 years) (median (range)) (4.0 (0.6–22.1) pmol/L) (*n* = 120) (*p* < 0.0001). We also found a slightly higher median IF concentration in men (median (range)) (4.9 (1.7–22.1)) (*n* = 120) compared with women (median (range)) (4.1 (0.6–13.8) pmol/L) (*n* = 120) (*p* = 0.02). However, these differences in age and sex are small in absolute concentrations and judged not to be of clinical relevance.

**Figure 3:**
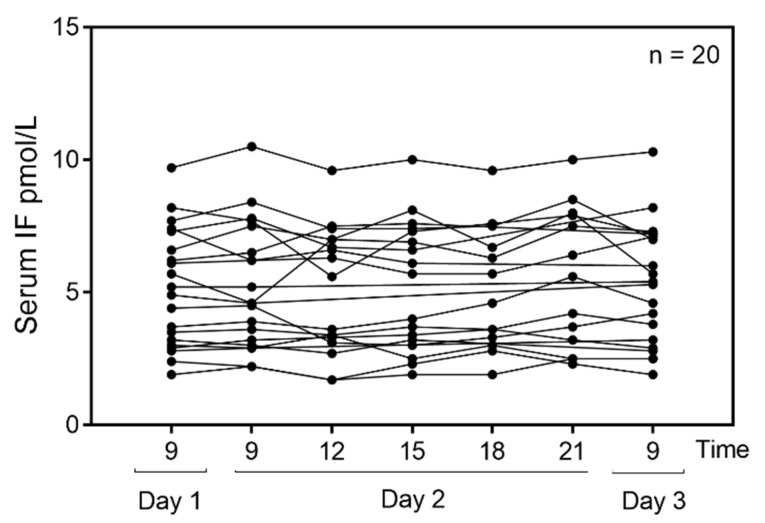
Diurnal variation of serum IF in healthy subjects. Serum IF was measured at different time points three days in a row in 20 healthy individuals. The 24-h clock format is used to indicate time of day. Out of 20 individuals, 13 donated blood at all time points and seven donated at some of the time points (all measures are shown in the figure). The RM-ANOVA was used to estimate differences in serum IF over time. No diurnal variation was found in this healthy cohort (*p* = 0.2).

To test for daily fluctuations in serum IF, we examined serum samples from 21 healthy subjects aged (median (range)) 39 (25–57) years (65% females). All but one showed a baseline IF concentration that was well within the established reference interval presented in Table 1 (median (range) for the group: 5.1 (1.9–9.7) pmol/L, *n* = 20). One subject, a 56 year old woman, showed a very high IF concentration (1600 pmol/L) and her data were excluded from the final statistical analysis. Due to ethical rules, it was not possible to obtain further information concerning this subject. The diurnal variations of serum IF (*n* = 20) are presented in Figure 3. We find no statistical variation over time (*p* = 0.2).

## 4. Discussion

We have established a sensitive ELISA for human IF and have shown that IF immunoreactivity is present in measurable amounts in human serum. Furthermore, we have established a 95% reference interval for serum IF in a population of healthy Danish blood donors, and have shown that the serum IF immunoreactivity concentration does not fluctuate during the day.

The finding of IF immunoreactivity in the circulation supports our hypothesis that gastric IF production leads to a small retrograde secretion of IF into the blood, in a manner that may be similar to that of salivary amylase [4]. This hypothesis could be explained by the findings of Howard et al. (1996) that IF expression is not restricted to the parietal cells of the stomach, but may also occur at the margins of the gastric regions [14]. Until now, no one has indisputably identified IF in the circulation, possible due to the lack of an assay sufficiently sensitive to detect minute amounts of the protein. We demonstrate the presence of IF immunoactivity, but obviously do not prove the existence of an IF-B12 complex in the circulation.

The established ELISA allowed for quantification of IF immunoreactivity down to 0.2 pmol/L with an overall intra-assay imprecision of 7.9% and a total imprecision of 15%. Notably, no alterations of IF occurred upon repeat freeze-thawing cycles, nor upon storage of samples for up to 33 months. Thus, the assay may prove very useful also for analyzing archival serum samples.

We consider our assay to be highly specific for IF as judged by size exclusion chromatography and the lack of cross reactivity with the B12-binding proteins, HC and TC, that are structurally related to IF [15] and occur in nanomolar concentrations in serum [1].

We examined the concentration of IF immunoreactivity in serum from healthy individuals and established a 95% reference interval (1.7–11.5 pmol/L). No difference in IF concentrations was observed between the sex, but a slightly higher IF concentrations was observed for middle-aged individuals (41–65 years) compared with younger (18–40 years). We speculate that, since the gastric mucosa becomes more permeable with age [15,16], this could be an underlying cause for this minor difference. Another possibility is that the slightly higher serum IF in older people is caused by an age-reduced kidney function [16] or age-related autoimmune gastritis [17]. Studies in the elderly (>65 years) may help clarify this matter.

We did not find any fluctuation in serum IF during the day. This is surprising, as the secretion of IF into the gastric lumen is expected to vary considerably [18] and may possibly have been overlooked, since only three samples were removed over 24 h.

## 5. Conclusions

In conclusion, we have presented a quantitative assay for measurement of human IF immunoreactivity in serum and established an interval of reference for healthy adult subjects. Our IF ELISA may prove useful for exploring physiological and pathophysiological aspects of the appearance of IF immunoreactivity in the blood stream.

## 6. Patents

E.G. and E.N. are inventors of a patent application (TECH-2018-631-115) filed by Aarhus University, Denmark, concerning this IF ELISA and its use for diagnostic purposes. Xeragenx has the first right of refusal.

## Figures and Tables

**Table 1:** Reference intervals for IF in serum.

Groups	*n*	2.5th–97.5th Percentile (90%CI)
All (18–65 years)	240	1.7 (0.9; 1.9)–11.5 (10.0; 16.5)
Men (18–65 years)	120	1.8 (1.7; 2.0)–13.8 (10.4; 22.1)
Women (18–65 years)	120	1.4 (0.6; 2.1)–10.2 (7.7; 13.8)
Young (18–40 years)	120	1.4 (0.6; 1.9)–10.9 (8.6; 22.1)
Middle-aged (41–65 years)	120	1.8 (1.7; 2.6)–13.3 (10.0; 13.8)

## Data Availability

Not applicable.
